# Projection Neuron Axon Collaterals in the Dorsal Horn: Placing a New Player in Spinal Cord Pain Processing

**DOI:** 10.3389/fphys.2020.560802

**Published:** 2020-12-21

**Authors:** Tyler J. Browne, David I. Hughes, Christopher V. Dayas, Robert J. Callister, Brett A. Graham

**Affiliations:** ^1^School of Biomedical Sciences and Pharmacy, Faculty of Health and Medicine, University of Newcastle, Callaghan, NSW, Australia; ^2^Hunter Medical Research Institute (HMRI), New Lambton Heights, NSW, Australia; ^3^Institute of Neuroscience Psychology, College of Medical, Veterinary and Life Sciences, University of Glasgow, Glasgow, United Kingdom

**Keywords:** pain, spinal cord – spinal cord connection, projection neurons, spinal circuits, sensory processing

## Abstract

The pain experience depends on the relay of nociceptive signals from the spinal cord dorsal horn to higher brain centers. This function is ultimately achieved by the output of a small population of highly specialized neurons called projection neurons (PNs). Like output neurons in other central nervous system (CNS) regions, PNs are invested with a substantial axon collateral system that ramifies extensively within local circuits. These axon collaterals are widely distributed within and between spinal cord segments. Anatomical data on PN axon collaterals have existed since the time of Cajal, however, their function in spinal pain signaling remains unclear and is absent from current models of spinal pain processing. Despite these omissions, some insight on the potential role of PN axon collaterals can be drawn from axon collateral systems of principal or output neurons in other CNS regions, such as the hippocampus, amygdala, olfactory cortex, and ventral horn of the spinal cord. The connectivity and actions of axon collaterals in these systems have been well-defined and used to confirm crucial roles in memory, fear, olfaction, and movement control, respectively. We review this information here and propose a framework for characterizing PN axon collateral function in the dorsal horn. We highlight that experimental approaches traditionally used to delineate axon collateral function in other CNS regions are not easily applied to PNs because of their scarcity relative to spinal interneurons (INs), and the lack of cellular organization in the dorsal horn. Finally, we emphasize how the rapid development of techniques such as viral expression of optogenetic or chemogenetic probes can overcome these challenges and allow characterization of PN axon collateral function. Obtaining detailed information of this type is a necessary first step for incorporation of PN collateral system function into models of spinal sensory processing.

## Role of PNs in Pain Signaling

### The Dorsal Horn and Ascending Pain Pathway

While it is widely appreciated that acute pain serves important biological functions, persistent or ongoing pain serves no apparent biological purpose, but has far-reaching impacts on patients and society. As for other sensory modalities, such as touch, heat/cold, and itch, we have a good understanding of the anatomy of the pathway over which noxious (or potentially painful) signals generated in skin, muscle, joints, and viscera travels to the brain *via* a series of synaptic connections on neurons located in various CNS regions or “pain nodes.” These nodes are located in anatomically discrete regions and, importantly, pain perception can be dramatically altered depending on the way an incoming sensory signal is processed (or passed on) at these nodes. Much effort has therefore focused on understanding how sensory signals are processed within the different nodes of the ascending pain pathway. This is especially the case for the first central node: i.e., the dorsal horn of the spinal cord. Here, signals carried on peripheral afferents combine with inputs from local interneurons (INs) and descending brainstem centers to determine whether information is relayed to higher brain centers. The reception of this information produces the sensory and emotional components of experience, we call pain (reviewed in more detail in: Todd, 2010; Peirs and Seal, 2016). The relay of nociceptive information from the spinal cord dorsal horn to higher brain centers is ultimately achieved by the output and axon of specialized neurons called projection neurons (PNs). This review focuses on what we believe is an overlooked element in dorsal horn pain processing, namely the role of PN axon collaterals that branch and terminate locally. These collaterals must share PN output with other dorsal horn neurons; however, their impact remains to be determined.

### PN Anatomy, Projections, and Synaptic Inputs

As the sole output cell of the dorsal horn, PNs have long been recognized as a vital element in spinal pain processing. Classically, they are considered to reside in laminae I and IV–V of the dorsal horn, with superficial and deep PNs being involved in nociceptive specific signaling or more broadly tuned signals that span innocuous to noxious ranges (wide dynamic range), respectively (Christensen and Perl, 1970; Coghill et al., 1993; Craig, 2003; Wercberger and Basbaum, 2019). Recent work in rodents, whereby PNs are labeled *via* retrograde tracer injections in the brainstem and thalamus, have provided more detailed information on the location of PN somata in the dorsal horn. PNs are concentrated in lamina I and the lateral spinal nucleus, absent in lamina II, and scattered throughout deeper laminae III–VI (Al-Khater et al., 2008) and lamina X (Krotov et al., 2017). Importantly, PNs are not a frequently encountered cell type in the dorsal horn. They make up <5% of the neurons in laminae I, 1% of the neurons in the superficial dorsal horn (laminae I–II) and are only sparsely scattered in the deeper laminae (Spike et al., 2003; Polgar et al., 2010; Cameron et al., 2015). Their scarcity alone has made PNs difficult to study, and this has hindered our efforts to understand how they integrate with other dorsal horn circuits (Brown, 1982).

The axons of PNs that transmit cutaneous pain, along with thermal and itch modalities, leave the spinal cord by traveling ventrally, crossing to the contralateral cord within a few segments, and finally ascending in the anterolateral tract (Todd, 2017). Transmission of pain associated with deep structures (e.g., viscera) ascends to the brain in either the anterolateral tract or dorsal column pathways (Willis et al., 1999). Textbook accounts have the axons of PNs terminating in the thalamus before being relayed to somatosensory cortex and limbic structures where the pain percept and its emotional or effective responses are generated. While this holds true for primates, recent work in rodents, however, has provided evidence for extensive PN axon terminals in brain stem centers, such as the nucleus of the solitary tract, caudal ventrolateral medulla, parabrachial nucleus (PBN), and the periaqueductal gray (Al-Khater and Todd, 2009; Cameron et al., 2015).

### PN Function in Pain Processing

**Figure 1:**
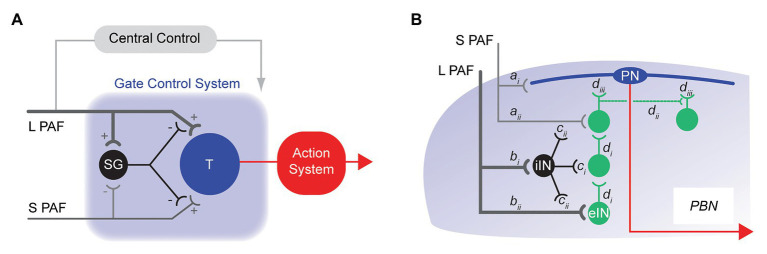
Historical models of spinal pain processing. **(A)**, schematic shows the originally proposed circuitry for the Gate Control Theory of Pain. Touch related sensory information is relayed to the dorsal horn by large fiber (myelinated) primary afferents (L-PAF), whereas nociceptive inputs are relayed by small fiber (unmyelinated) primary afferents (S-PAF). The transmission cell (T) represents the output cell [projection neuron (PN)] of the dorsal horn. An inhibitory interneuron (iIN) is key to the model and is represented as SG. Activation of the SG cell by L-PAF suppresses T cell activation, thereby reducing (gating) pain signaling (i.e., action system). Alternatively, S-PAF activation inhibits SG cell activity, facilitating pain signaling output from the T cell (action system). **(B)**, schematic summarizing more modern views of spinal pain processing circuits. Nociceptive information (S-PAF) is relayed into the superficial dorsal horn and terminates on lamina I PNs (*a_i_*) and local interneurons (*a_ii_*), whereas touch related sensory information (L-PAF) terminates in the deep dorsal horn (*b_i_* and *b_ii_*). Polysynaptic circuits of excitatory interneurons (eINs) circuits (*d_i_*) provide a channel for L-PAF input to excite PNs in lamina I (*d_iii_*). Excitatory interneurons also form polysynaptic networks (*d_ii_*) that can enhance nociceptive signaling onto lamina I PNs. Populations of iINs also receive sensory signals from the periphery and suppress the activity of nociceptive and touch related circuits *via*: postsynaptic inhibition of these circuits, and presynaptic inhibition of sensory inputs (*c_i_* and *c_ii_*, respectively). Supra-threshold excitation causes transmission of information along the PN axon (red line) to brainstem and midbrain structures, such as the parabrachial nucleus (PBN).

Our current understanding of how the spinal cord relays nociceptive signals from the dorsal horn to higher brain centers is still heavily influenced by the gate control theory of pain (Figure 1A) published in 1965 (Melzack and Wall, 1965). Although many of the fine details in this theory have since been refined and modified, at its heart is the premise that functional inhibition is critical for appropriate spinal sensory processing and normal pain perception (Yaksh, 1999; Zeilhofer et al., 2012; Mendell, 2014). Specifically, the gate control theory assigned inhibitory INs (iINs) a role in preventing touch inputs from activating pain circuits. More recently, a role for excitatory INs (eINs) in pathological pain has been added where polysynaptic pathways associated with touch information infiltrate nociceptive circuits (Neumann et al., 2008; Takazawa and MacDermott, 2010). Thus, IN networks in the dorsal horn are accepted as a critical factor in shaping output signals that are conveyed to the brain by PNs to produce pain. The final element identified in the original gate control theory is “central control,” where signals arising from the brain itself descend and modify spinal dorsal horn activity. Indeed, several brain structures are now well-established sources of descending input to the spinal dorsal horn (Suzuki et al., 2004; François et al., 2017; Lu et al., 2018). They have the capacity to facilitate or inhibit dorsal horn circuits and alter the gain of pain signaling in this region.

Building on the foundations established in gate control theory, our modern view of spinal sensory circuits in the dorsal horn still includes inhibitory gating mechanisms, with more recent transgenic technologies substantially advancing these views (Figure 1B). For example, termination zones are well-established for small diameter nociceptive primary afferents in the superficial dorsal horn and for large diameter tactile primary afferents in the deep dorsal horn (Peirs and Seal, 2016). We now know deep and superficial laminae are linked by polysynaptic circuits of eINs (Brown, 1982; Abraira et al., 2017). This provides a pathway for touch related sensory information to be relayed, or infiltrate, into lamina I and excite lamina I PNs, activating the ascending pain pathway. Populations of eINs have also been shown to form excitatory networks that support reverberating excitation in these circuits. Finally, iINs have been shown to act through a variety of connections to mediate presynaptic and postsynaptic inhibition and thus regulate, or gate, spinal sensory processing. In summary, our current understanding of how nociceptive signals are processed in the dorsal horn is built on the interaction of *three distinct factors* (sensory afferents, dorsal horn INs, and descending brain pathways). Together, the summed activity of these elements determines PN outputs that evoke the pain experience in the brain. We propose PN axon collaterals represent a missing *fourth factor* that must be incorporated into current models of spinal pain processing.

## Axon Collateralisation in the Dorsal Horn of the Spinal Cord

The anatomy and function of PNs have been studied extensively for over 60 years (Wercberger and Basbaum, 2019) as they play such a key role in channeling highly processed information from the dorsal horn to supraspinal targets. This view is supported by several studies that show a dorsal flow of sensory signals through anatomically and functionally connected dorsal horn IN populations to PNs that can then relay this information to the brain (Cordero-Erausquin et al., 2009; Hachisuka et al., 2018; Smith et al., 2019). The fact that PNs project to readily accessed brainstem centers such as the PBN and periaqueductal gray has facilitated their identification and study in spinal cord slices through retrograde labeling approaches (Ruscheweyh et al., 2004; Dahlhaus et al., 2005). As a result, we now have some information on the discharge properties (Ruscheweyh et al., 2004), and involvement in certain forms of synaptic plasticity (Ikeda et al., 2006). Few studies have examined PN axon collaterals, even though recent data (see below) show they provide a substrate for pain signals destined for the brain to be shared with, or “copied to” circuitry within the spinal cord. The phenomenon termed efference copy in other systems (Sperry, 1950).

### Anatomical Evidence for Axon Collaterals in the Dorsal Horn

**Figure 2:**
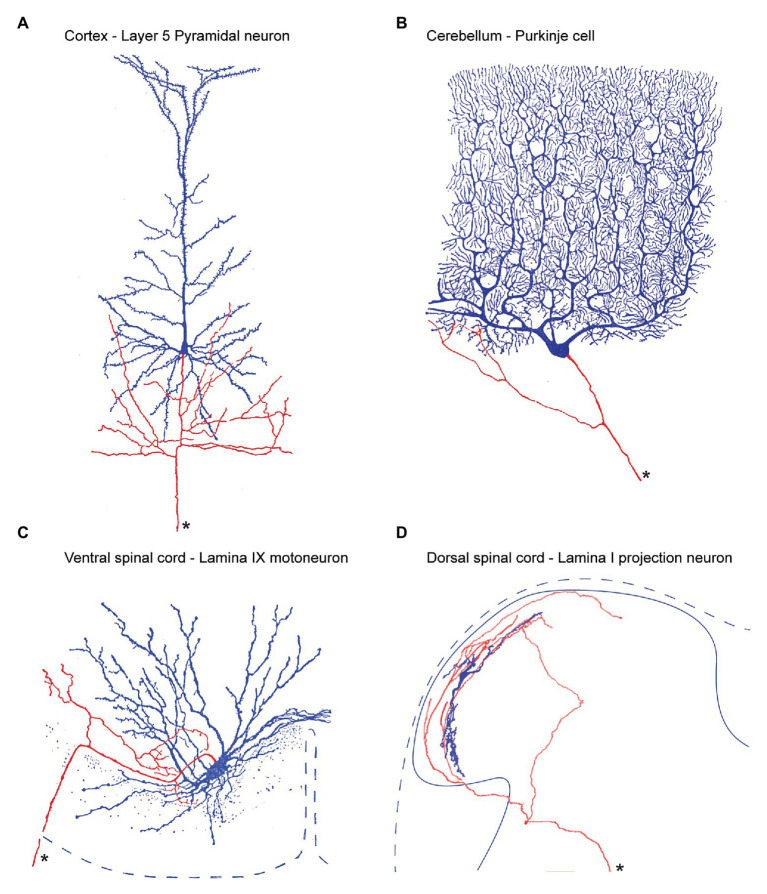
Output neuron axon collateral anatomy throughout the central nervous system. **(A)**, within the cerebral cortex, Layer 5 Pyramidal neurons provide the principle output which can project to regions as distant as the lumbar spinal cord, to neighboring cortical regions, or other brain regions such as the thalamus. In addition, Pyramidal axons branch locally producing an extensive local collateral network (soma and dendrites labeled blue, axon labeled red, and main axon marked by an asterisk). **(B)**, Purkinje cells are the output cells of the cerebellar cortex and send axons to deep cerebellar nuclei. Purkinje cell axons also branch to produce a local collateral network **(C)**, Motoneurons are the output cell of the ventral spinal cord. Their long axons exit the ventral spinal root and innervate skeletal muscle. In addition, motoneuron axons branch within the ventral horn to produce an extensive local collateral network. **(D)**, Lamina I PNs are the output cell of the dorsal spinal cord. Their main axon crosses the midline and ascends to various regions including the PBN, and periaqueductal gray, and thalamus. Lamina I PNs also exhibit an extensive local collateral network. Panels **(A–C)** modified from (Cajal, 1952) and **(D)** is from (Szucs et al., 2010).

Local branches and collaterals arising from the axon of principle (output) neurons are a feature of many CNS regions, including the cortex, cerebellum, and ventral horn of the spinal cord (Figure 2). For dorsal horn PNs, most information we currently have on local axon collaterals comes from electrophysiological studies on identified PNs, where various labels are included in intracellular recording electrodes. Reconstructions of filled PNs have documented axon collaterals in monkey (Beal et al., 1981), cat (Bennett et al., 1981; Brown, 1981; Hylden et al., 1985, 1986; Maxwell and Koerber, 1986; Light et al., 1993), and rat (Luz et al., 2010; Szucs et al., 2010, 2013). Surprisingly, no data are yet available for PN axon collaterals in mouse, despite the increased use of genetically modified mice for pain studies.

Szucs et al. (2010) have provided the most detailed characterization of PN collaterals within lamina I of the rat dorsal horn. They used an *ex vivo* intact spinal cord preparation (lumbosacral enlargement, L1–L6), which allows visualization and identification of presumptive PNs based on their superficial location, large size, and the *post hoc* tracing of filled-cell axons tracking to the anterolateral tract. Notably, data using this approach showed PN axon collateral territories were extensive and confined to the ipsilateral cord. PN axon collaterals could be classified into four types according to their course and territory in the dorsal horn. Dorsal collaterals ramified extensively within laminae I–IV of the dorsal horn gray matter and sometimes entered Lissauer’s tract. Ventral collaterals projected into the propriospinal and ventral motor territories (Flynn et al., 2011). Lateral collaterals ran primarily in the rostrocaudal plane for about a spinal segment. Finally, mixed collaterals exhibited combinations of the other three types. Importantly, in all four types, en-passant and terminal synaptic boutons were observed on PN axon collaterals – implying the potential for local (spinal) signaling. The authors concluded that PN axon collaterals likely have an important function in local circuits including intra- and intersegmental spinal cord processing. Furthermore, this widespread distribution positions the ascending signals carried by PN collaterals to influence the spinal circuits that encode pain, itch, touch, visceral pain, as well as proprioception and motor output.

### Neurochemical Phenotype and Function of PN Axon Collaterals

The Todd group (Cameron et al., 2015) showed *via* retrograde labeling and immunohistochemistry that 97% of the synaptic boutons on PN axons in the PBN expressed VGLUT2. This matches the expected excitatory nature of PNs. Presumably, this would also apply to the *en passant* synaptic boutons on PN collaterals within the spinal cord. vGLUT2 is also detected in the majority (>85%) of the output cells in the medullary dorsal horn (Ge et al., 2010; Zhang et al., 2018). Assuming conservation between the spinal and brainstem dorsal horns, these observations further support the likelihood that spinal cord PN collaterals mediate excitatory actions on their immediate, local targets. While the case for glutamate signaling is clear, this work does not exclude the potential for PN terminals to also release neurotransmitters other than glutamate. For example, the same work that confirmed VGLUT2 expression in PN axons also reported some terminals expressed Substance P (~16%). Furthermore, precedent for co-transmission can be found in the ventral horn of the spinal cord, where motoneurons have been shown to utilize multiple neurotransmitters (acetylcholine and glutamate) at synapses in their axon collaterals that excite Renshaw cells (Renshaw, 1946; Callister and Graham, 2010; Lamotte d’Incamps et al., 2017; Callister et al., 2020). Thus, the available anatomical data suggest extensively ramified PN axon collaterals can make excitatory synaptic connections with neurons in the dorsal, intermediate, and ventral horn; however, the functional role of additional transmitters (e.g., peptides) remains to be established.

To the best of our knowledge, there is only one demonstration of a functional synaptic connection made by a PN axon collateral within dorsal horn, albeit from a putative PN in the young rat spinal cord. In this example, Luz et al. (2010) used paired patch clamp electrophysiology and recorded a monosynaptic excitatory connection between two putative PNs. It was not reported if the connection was strong enough to generate a spike in the post-synaptic PN; however, inputs from other lamina I INs were capable of generating spikes in PNs. These data, coupled with the anatomical data presented above, strongly suggest PN axon collaterals have an active role in the dorsal horn of the spinal cord. Substantially, more information will be required; however, before PN axon collateral signaling can be incorporated into models of spinal sensory processing.

## Axon Collateral Function in Other CNS Regions

Since the time of Cajal, neuroscientists have found it useful to organize the elements that make up various CNS regions into three components: afferent inputs that bring information to a region, INs that are involved in local processing, and principal/relay/output or PNs that transmit processed signals. The later sends out a long axon that carries information to another CNS region or peripheral target. This model also acknowledges a role for local collaterals arising from output neurons (e.g., “the principal or output neuron can also take part in local processing and does so by axon collaterals that ramify extensively among local circuit neurons” Shepherd, 2004).

While the roles of dorsal horn PN axon collaterals remains unknown, the function of such recurrent circuitry has been clarified within other CNS regions. For example, the Schaffer-collateral system in hippocampus, composed of axon collaterals of CA3 pyramidal cells, forms an integral part of the trisynaptic pathway, and the synapses they form onto CA1 pyramidal cells are the most widely studied synaptic connections of any within the central nervous system (Freund and Buzsáki, 1996). In the following section, we discuss some of these PN-derived feedback circuits, and by highlighting common motifs among these examples, provide insight into the potential functions within the spinal dorsal horn.

**Figure 3:**
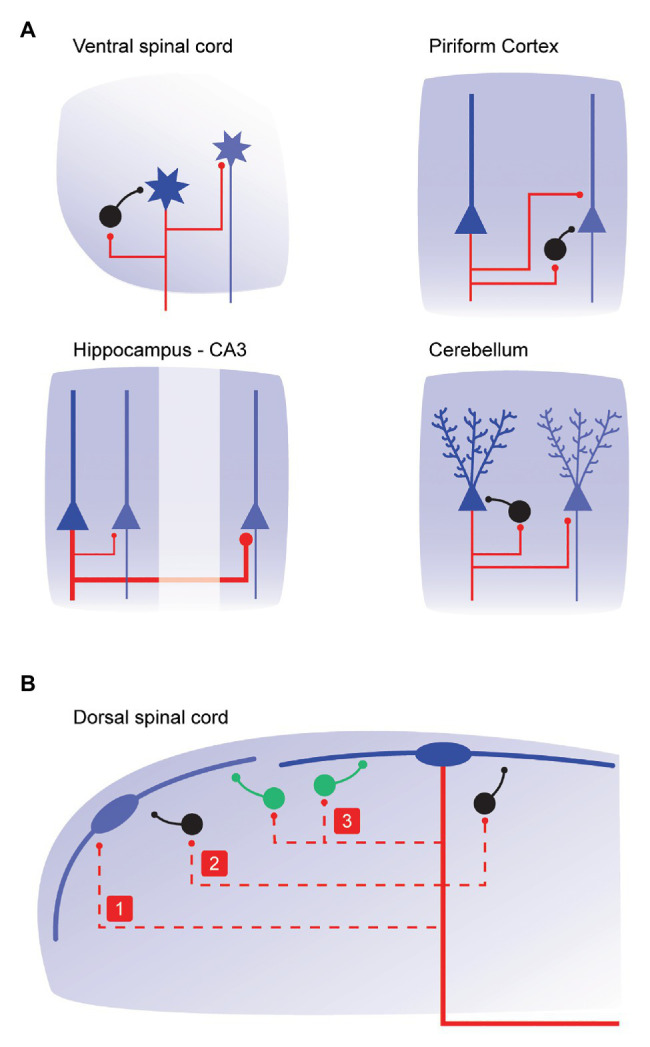
Connectivity and function of local axon collateral networks throughout the central nervous system. **(A)**, schematics summarizing axon collateral connectivity in CNS regions where their function is well-characterized. Primary output neurons in each region are shown in blue, the axons and collaterals from these cells are shown in red, and local iINs are black. Local axon collaterals arising from motoneurons in the ventral horn (upper left) contact inhibitory Renshaw cells and neighboring motoneurons. Functionally, these connections are implicated in varying force and in rapidly increasing force, respectively. In the piriform cortex (upper right), local axon collaterals arising from pyramidal neurons contact local iINs and other pyramidal interneurons. These connections are critical for stable invariant odor coding regardless of concentration, and recruitment of stable populations of Pyramidal neuron including cells that may not receive primary odor inputs. In the CA3 region of the hippocampus (lower left), pyramidal neuron axon collaterals contact other pyramidal neurons with a distal to proximal density (axon collateral thickness is proportional to synaptic drive). This allows the two regions to serve different roles. The collateral innervation of more proximal CA3 neurons is crucial for memory storage (pattern recognition), whereas distal CA3 contacts are important for memory retrieval (pattern completion). Finally, Purkinje cell axon collaterals in the cerebellum (lower right) contact local iINs and other Purkinje cells to provide inhibitory feedback and form feedforward loops. These are important for restricting local activity to the cerebellum’s well-defined parasagittal zones. **(B)**, schematic shows local axon collateral connections (red dashed lines) that could arise from lamina I dorsal horn PNs (blue). Based on collateral connectivity summarized in **(A)**, PN collaterals in the dorsal horn may contact: (1) neighboring PNs; (2) local iINs; or (3) local excitatory interneurons (green). These connections may be relevant for shaping intensity, receptive field properties, and the modality of PN output that is ultimately relayed to the brain.

Our best understood example of axon collateral function in the spinal cord stems from studies of “recurrent inhibition” in the ventral horn of the spinal cord (Figure 3A). As early as the 1940s, there was compelling anatomical evidence for the existence of motoneuron axon collaterals. Importantly, these collaterals terminated in the vicinity of other ventral horn cells. The function of this collateral system was initially studied by Birdsey Renshaw in a series of experiments over 1938–1941 (see Callister et al., 2020). Renshaw showed that “reflex” motoneuron activity, initiated by stimulating dorsal roots or dorsal columns, was inhibited by stimulating cut ventral roots (Renshaw, 1941). He proposed that motoneuron axon collaterals produced inhibition in the ventral horn. Eccles subsequently studied the important inhibitory neuron that was underpinning the observation, termed the Renshaw cell (Eccles et al., 1954). It represents one of the first neural circuits ever described in the CNS, and by extension the first to involve axon collateral signaling.

In its simplest form, activation of the motoneuron and its axon collateral, results in the recruitment of the Renshaw cells. In turn, these Renshaw cells then inhibit the homonymous motoneuron pool with glycinergic inhibition. This feedback is not trivial, and evidence has shown that input from one Renshaw cell can cease activity in its coupled motoneuron (Bhumbra et al., 2014). This coupling arrangement has been viewed as a variable gain regulator that allows muscles to produce a wide range of contractile forces (Moore et al., 2015). It is also thought to be important in preventing the runaway excitation of the motoneuron, acting to enable smooth motor control and prevent muscle spasticity such as in *Clostridium Tetani* infection (tetanus). More recently, motoneuron axon collaterals have been shown to target neighboring motoneuron (Spike et al., 2003; Bhumbra and Beato, 2018). Collateral input is to both local (intrasegmental) and more distal (intersegmental) motoneurons and is thought to play a role in progressively recruiting motor neurons to rapidly increase muscle force. As previously noted, PN collaterals in the dorsal horn are similarly arranged in both inter- and intrasegmental territories. This suggests they could provide recurrent inhibition to PNs as well as recruiting neighboring PNs. The capacity of the motoneuron circuit to both alter signaling range and rapidly increase the intensity of ventral horn outputs implies PN axon collaterals may help tune the boundaries of sensation and the intensity of pain signals that are ultimately transmitted to the brain.

In the brain, output neurons in the cerebral cortex, hippocampus and amygdala also give rise to extensive local axon collaterals and, in these regions, much is also known about function. In the olfactory (piriform) cortex, axon collaterals form a sparse excitatory network among output “pyramidal” neurons (Figure 3A). This connectivity is thought to allow odors to recruit ensembles of pyramidal neurons including cells that do not directly receive an odor input (Franks et al., 2011). Pyramidal neuron collaterals also activate feedforward and feedback inhibition, *via* local INs. The latter is critical for stable “invariant” odor coding, regardless of changes in odor concentration (Bolding and Franks, 2018). In the basolateral nucleus of the amygdala, principal cell collaterals show spatial selectivity whereby iINs are contacted by terminals on proximal collaterals, and, other principal cells are contacted by those on more distal collaterals (Duvarci and Pare, 2014). This configuration is considered important to prevent runaway excitation within the primary cell, while allowing associative interactions with more distal principal cell populations that underpin associative conditioning (such as fear). In the hippocampus, the Schaffer-collateral system plays a critical in memory processing (Figure 3A). Specifically, graded proximal to distal collateral density along CA3 allows proximal CA3 to perform important roles in responding to novel cues with a tendency to remap and store new memories (i.e., as in pattern recognition). Alternatively, distal CA3 with extensive collaterals is thought to drive memory retrieval functions (pattern completion), especially with degraded or incomplete input patterns (Lee et al., 2015). A similar associative excitatory collateral network has also been described for grid cells in medial entorhinal cortex. This is thought to be important for the correct neural representation of space (Couey et al., 2013). Extending these observations to spinal sensory processing in the dorsal horn, a similar excitatory collateral network connecting PNs, coupled with collateral connectivity to iINs could easily play a role in the reliable representation of sensory signals from the body surface (i.e., receptive fields). Furthermore, in terms of pain signaling, this arrangement would allow for receptive field expansion as observed following tissue damage (Figure 3B).

A final example of collateral signaling highlights that these output neuron configurations are not always excitatory. In the cerebellar cortex, the major output neuron, the Purkinje cell, is inhibitory. Various models of cerebellar function have existed for years (Eccles et al., 1967) based on a feedforward circuit with synaptic activity flowing from mossy fibers through granules cells to the Purkinje cell. These neurons have an extensive axon collateral system that is organized preferentially in the parasagittal plane (Watt et al., 2009). Recent advances have allowed detailed functional investigation of Purkinje cell collaterals in adult rodents. Witter et al., 2016, have shown these collaterals provide inhibitory feedback to several classes of INs and also contact neighboring Purkinje cells (Witter et al., 2016). These important feedback loops are considered important for regulating activity in the cerebellum’s well-known parasagittal zones. Thus, even when collaterals arise from inhibitory population’s they have been shown to play a role in delineating functional borders and confining excitation.

In summary, there are precedents for axon collaterals playing important roles in the function of various CNS regions *via* either excitation or inhibition (Figure 3A). Generally, these output neuron collaterals drive feedback inhibition through local INs or have amplifier and associative functions *via* feedforward excitation. Such data are not yet available for PN axon collaterals, however, insights from other collateral systems throughout the CNS suggest that new information on PN axon collaterals will have a major impact on the way we understand spinal sensory processing (Figure 3B).

## New Techniques and a New Approach to Study PN Axon Collaterals

Two major factors have likely hampered our understanding of PN axon collateral signaling in the dorsal horn, compared to the relatively advanced understanding of collateral signaling in other regions. First, the scarcity of PNs in the dorsal horn (~1% of lamina I–II neurons vs. 70–90% principle neurons in cortex, hippocampus, and amygdala) makes them difficult to target with a microelectrode. Second, a definitive genetic characteristic or “signature” that distinguishes PNs from other dorsal horn cells remains to be identified. This contrasts with ventral horn motoneurons, for example, that can be easily identified by location (Lamina IX) and cholinergic markers.

A series of recent technical advances now permit progress in understanding the role PN axon collaterals in spinal sensory processing circuits. Specifically, viral gene transduction with specific trafficking and tropism properties allows gene targeting and protein expression to be directed to neuronal populations according to where their axons ultimately project. This is similar to the classical retrograde labeling approaches that involved injection of tracers, such as HRP, WGA, fast blue, and fluorescent microspheres, into brain regions, such as PBN, periaqueductal gray, thalamus, or rostroventromedial medulla. Application of modern gene targeting and protein expression approaches results in robust expression of fluorescent proteins (e.g., GFP, mCherry, and TdTomato). This allows visualization of dendritic and axonal processes to an extent not possible using classical tracers. Furthermore, multi-labeling experiments using brainbow constructs, a genetic cell-labeling technique that identifies individual cells with unique color hues (Weissman and Pan, 2015), will allow mapping of the relationship between populations of dorsal horn PNs in great detail.

Viral approaches also present an opportunity to express proteins that allow control of PN activity *via* optogenetic or chemogenetic techniques, both *in vitro* and *in vivo*. These methods are particularly relevant to our efforts to study PN axon collateral function in the dorsal horn. For example, channelrhodopsin-2 assisted circuit mapping (CRACM) could be achieved by selective photostimulation of PNs in acutely prepared tissue slices. The CRACM approach has been used to functionally characterize and map axon collateral connections of semilunar cells (a specific type of principle/output neuron) in piriform cortex. Optogenetic activation of semilunar cells produced recurrent excitation of collaterals that contacted a different population of output cells, termed superficial cells (Choy et al., 2017). CRACM has also been used to study the targets of Purkinje cell axon collaterals in the cerebellum. Notably, optogenetically identified connections contacted other Purkinje cells, molecular layer INs, and Lugaro INs, but *not* Golgi INs (Witter et al., 2016). These results highlight how the optogenetic approach can uncover functional collateral connectivity patterns that would take years to acquire using the low-yield and technically demanding paired patch clamp recording approach.

Transgenic manipulations that silence synaptic function have also been applied while using *in vitro* slice electrophysiology, as well as in anesthetized and freely behaving animals to study local axon collateral function. For example, work in the piriform cortex has used transgenic expression of tetanus toxin light chain (TeLC) to silence pyramidal cell axon collateral synapses while retaining the intrinsic excitability of these cells (Bolding and Franks, 2018). This approach confirmed local collateral signaling that recruited feedback iINs was critical for tuning the local response to concentration-invariant coding of odors. The same approach has also been used to silence the recurrent excitatory circuits of CA3 pyramidal neurons and assess the collateral network’s role in epilepsy (Yu et al., 2016). This manipulation reduced kainic acid-induced seizure activity, hippocampal epileptiform oscillatory activity, and cFos expression. This confirmed the long-held view that CA3 pyramidal neurons, and their extensive axon collateral networks, are critical for kainic-acid triggered seizures. These two examples highlight how transgenic targeting of axons and synaptic function allows axon collateral function to be comprehensively studied. Similar approaches in the dorsal horn may help clarify the importance of local PN axon collaterals in sensory experience, although the anatomy of the nociceptive pathway does present some challenges. Specifically, TeLC silencing would prevent local collateral signaling as well as silencing the ascending connections that rely nociceptive signals to the brain. Thus, it may be difficult to attribute any behavioral phenotype to a specific connection type. Despite this, the proximity of local collateral synapses to the PN cell body, versus the distant projections synapsing in the brain, may be advantageous. This could open a window whereby high local (spinal) TeLC expression achieves local silencing, before significant TeLC expression reaches the more distal brain connections. This delay could allow the direct assessment of PN derived recurrent feedback in the dorsal horn while maintaining normal neuronal excitability and upstream transmission of nociceptive signals.

## Conclusion and Future Directions

The literature reviewed above provides clear evidence that a local axon collateral system exists in the spinal dorsal horn arising from its output cell, the PN. The only functional information on PN axon collaterals in the dorsal horn comes from a single observation at a connection between two putative PNs in tissue from a young animal. No other functional information is available on these connections. A more advanced understanding of local axon collateral function in other CNS regions suggests “feedforward” excitation of other PNs, and recurrent “feedback” inhibition through connections with iINs is likely to exist. Fortunately, the experiments required to clarify these proposed roles are now possible because of new transgenic and viral techniques that can be applied in the mouse. Accordingly, we suggest the following pressing questions can and need to be answered:

1. Do mouse dorsal horn PNs exhibit branching and local axon collaterals? Anatomical evidence for this and the existence of synaptic specializations has been provided in other species, but not mouse.
2. Do dorsal horn PNs that reside outside laminae I give rise to collateral systems and are they similar? The most detailed characterization, available in rat, only reported on axon collaterals arising from superficial lamina I PNs that could be visualized in an *ex vivo* intact spinal cord preparation.
3. Which type of dorsal horn neuron do PN axon collaterals target? As outlined above, three broad dorsal horn populations include PNs, eINs, and iINs.

As answers to these questions become available it will be possible to incorporate PN axon collateral signaling into models of spinal sensory processing and thereby include an important new player in these circuits. While it is not yet possible to definitively predict how this information will change our understanding of spinal pain processing, available evidence from other output neurons suggests sharing the ascending “output” pain signal with local spinal pain circuits will be important. This information needs to be first collected under naïve uninjured conditions. Future experiments can then ask whether dysfunctional collateral signaling exists in pain models (both neuropathic and inflammatory). Finally, if this detailed information identifies a discrete post-synaptic arrangement of PN axon collaterals, for example capable of blunting pain signals (such as the Renshaw cell), it will represent an attractive foundation from which to develop targeted, and selective new pain therapies.

## Author Contributions

All authors contributed equally to all planning, writing and editing associated with this invited submission to a Frontiers Research Topic.

### Conflict of Interest

The authors declare that the research was conducted in the absence of any commercial or financial relationships that could be construed as a potential conflict of interest.
